# Firefighters and police search dog handlers’ experiences working closely with paramedics in urban search and rescue incidents: a qualitative focus group study from Oslo

**DOI:** 10.1186/s13049-024-01194-1

**Published:** 2024-03-19

**Authors:** Erik Westnes, Magnus Hjortdahl

**Affiliations:** 1 Ambulance department, Division of Prehospital Services, Oslo University Hospital, Oslo, Norway; 2 Department for Prehospital Education and Research, Faculty of Health Sciences, Oslo Metropolitan University, Oslo, Norway; 3 Department for Emergency Medical Coordination Centre, Division of Prehospital Services, Oslo University Hospital, Oslo, Norway

**Keywords:** USAR, Urban search and rescue, Inter-agency training, EMS, Trust-building in emergency services, Team-building in emergencies, High-risk emergencies

## Abstract

**Background:**

Rescue operations are in Norway defined as situations where patients are difficult to access or that more resources are needed than the health services alone possess and can put in operation (Bull A, Redningshåndboken er endelig her! [Internet]. Hovedredningssentralen. 2018 [cited 2023 May 15]. Available from: https://www.hovedredningssentralen.no/redningshandboken-er-endelig-her/). Rescue operations after large incidents may include civil protection, military forces, non-governmental organizations and other resources, but the initial rescue effort must be performed by the emergency services as time often is of essence. The central area of an accident where special training and personal protection equipment is necessary or mandatory is called the Hot Zone. This study examines Urban Search And Rescue (USAR) firefighters and police officers reported experiences from ambulance personnel’s contribution in the Hot Zone.

**Methods:**

We conducted five focus group interviews with USAR-trained firefighters and police officers. The interviewees were those on duty on the agreed dates. The interviews were taped, transcribed, and analysed using thematic analysis as described by Braun & Clarke.

**Results:**

Three themes were identified; Feeling safe during missions, Building USAR capacity, and Trust-building within USAR-teams. The firefighters and police officers reported their and the patients’ safety are best managed by EMS-personnel, whose presence strongly contributes to their own feeling of safety in a dangerous area. When EMS handles victims and injured emergency workers, firefighters and police officers can focus on their own primary tasks. Indeed, interviewees reported that building a USAR capacity depends on having USAR-trained EMS-personnel in the Hot Zone. The interviewees have clear and consistent opinions on how to establish an interagency USAR capacity effectively. Trust is paramount to the interviewees, and they express a high degree of trust within USAR Oslo.

**Conclusions:**

Firefighters and police officers regard USAR-trained EMS-personnel as a natural and integrated part in urban search and rescue teams. EMS-personnel in the dangerous area deliver safety and medical professional assistance to both rescue workers and patients. Informants in this study had clear opinions on how to establish and maintain such a service.

## Background

Urban Search And Rescue (USAR) refers to concepts and capacities to respond to complex and technical rescue operations. USAR has received massive media coverage in Norway due to the quick clay landslide in Gjerdrum December 30th 2020. Similarly, international emergencies have made USAR relevant in the public, emergency services, and among stakeholders, including the war in Ukraine where civilian infrastructure has been targeted daily, and the earthquake in Turkey and Syria February 6th 2023. 

When civilian infrastructure is exposed to force it is not built to withstand, collapse and inevitable human suffering must be expected. It does not matter much whether that force is due to extreme weather, climate change, industrial accidents, terror, or regular acts of war. “United Nations (UN) General Assembly Resolution 57/150 (December 16th 2002) identifies that each country has the responsibility first and foremost to take care of the victims of disasters and other emergencies occurring on its territory.” [2].

The de facto international standard for USAR is defined by the International Search And Rescue Advisory Group (INSARAG), an international network with more than ninety member countries, sorting under the UN Office for the Coordination of Humanitarian Affairs (OCHA). This standard is defined in guidelines for establishing USAR preparedness and a list of which capacities such a preparedness should comprise [2]. As of February 2024, Norway has not yet established an INSARAG-certified USAR capacity for responding to either domestic or international USAR incidents. While local initiatives among emergency services have established loosely connected and framed capacities, without governance and national regulations, these capacities are not possible to exploit to their fullest potential.

Beredskapsenheten (BE), a subunit of the ambulance department at Oslo University Hospital, developed and established a medical USAR capacity during a one-year period starting in 2017.

Oslo Police District’s (OPD) patrol dog unit (Victor) and Oslo fire and rescue service (OBRE) had already established a cooperation regarding USAR incidents two years prior, but they recognized they lacked the medical competence to manage both injuries to be expected in USAR-related rescue effort and necessary medical treatment to USAR-victims. This recognition was partially based on the experiences of NORSAR (Norwegian Search And Rescue) in the international humanitarian aid effort after the earthquake in Nepal [3] in 2015. NORSAR was comprised of USAR-trained firefighters from OBRE Special Rescue, non-USAR-competent doctors and paramedics from the Air Ambulance department at Oslo University Hospital, and USAR-trained search dogs and handlers from Victor. However, Norwegian authorities chose not to continue funding NORSAR after the Nepal incident. In the years after 2018, BE reported that they had educated and trained enough paramedics and EMTs to be prepared for USAR effort 24/7. Subsequently, Victor, OBRE, and BE named their combined USAR capacity “USAR Oslo”.

This study examines USAR Oslo’s experiences with having health professionals in the Hot Zone of USAR incidents, exercises, and training. Hot Zone in this respect is defined as the central part of an accident area where special training, protection equipment and gear is necessary or mandatory.

There are few empirical papers that discuss the actual experiences of emergency workers regarding inter-agency collaboration at the ground level. For example, a South African master thesis from 2016 [4] based on an international questionnaire, asked UHP (USAR Health Professional) several questions about their training in USAR-specific topics. While the findings are interesting, they are not directly relevant as they were based on questioning health professionals only about their perception of received education on USAR. Other publications address international humanitarian efforts on organizational and financial topics [3, 5], again, not directly relevant to the topic of this study.

In this study we examine USAR trained firefighters and police search dog handlers’ experiences with USAR trained EMS-personnel in USAR-incidents: How do USAR trained EMS-personnel contribute, and how can they offer even better assistance to both rescuers and victims?

## Methods and methodological considerations

This is a qualitative study, using focus group interviews [6, 7]. The thirty-two items on the COREQ [8] checklist have been followed with a few minor omissions, as documented below. Three interviews were conducted with USAR operators from OBRE and two with search dog handlers at Victor in Oslo, Norway. The aim was to collect experiences from actual USAR operators on collaborating with USAR trained EMS personnel, and if possible, find critical success factors and possible pitfalls. Magnus Hjortdahl (MH) works as an advisor at the Emergency Medical Call Centre (EMCC) at Oslo University Hospital and associate professor at the faculty of health sciences at OsloMet University. He has extensive experience in qualitative research, but no background or knowledge about USAR. Erik Westnes (EW) works as a paramedic in the air ambulance department at Oslo University Hospital with more than twenty years of prehospital experience both there and in the ambulance service. EW was central in establishing USAR capacity in the ambulance department.

### Data collection

MH conducted and tape recorded the interviews. After the first two interviews, the interview guide was reviewed and adjusted. After five interviews EW and MH found that the last two to a large degree repeated the same responses as the first three related to the research question, that is, they had reached a degree of saturation [7]. Preliminary analysis indicated that we had enough data to answer our research question, as we had started to come across the same patterns.

### Participants

The department managers and participants received a written information letter a fortnight prior to the interviews, and they all signed a concession agreement before the interviews started. No one withdrew their agreement during or after the interviews. One participant wanted to read the transcript of the interview and confirmed by email the transcript was correct.

Participants were everyone on duty on the dates selected by managers at the two services. EW and MH had no insight in or presented any selection criteria for interview subjects, only that they were USAR-trained within their service. MH conducted the interviews at their premises, at the start of their shifts. The numbers of participants were three in two interviews, four in the remaining three. These five interviews comprised about 50% of the employees in OBREs Special Rescue Unit and 30% in Victor, respectively. None participated in more than one interview. Their experience as USAR-operators ranged from six months to fifteen years, with an overall average of seven years. All participants took active part in the interviews.

### Analysis

EW transcribed the interviews and analysed them using thematic analysis as described by Braun & Clarke [9, 10] using NVivo 1.7.1 by QSR International. For further insight on the method, EW participated in the SAGE Publishing online webinar “Common challenges in Thematic Analysis and how to avoid them” with Braun & Clarke on August 11. 2022 [11]. According to the method, some codes and suggested themes were expected in advance, more found during listening to recordings, identified during transcription, and several more during active searching. Indeed, some themes were found as expected, but some that were expected were not found. Codes were registered in NVivo during listening and transcription, according to Braun & Clarke’s six step model [10]:

• Phase 1—familiarizing with the data

• Phase 2—generating codes (Table 1)

**Table 1:** Codes in phase 2

Name	Files	References
Area of responsibility	5	24
Authorities do not own USAR	2	12
Bottom up or top-down	2	4
Capacity	5	15
Common mental model	5	14
Competence	4	26
First aid	2	3
INSARAG or other certification	1	1
Know eachother's equipment	3	9
Know the face	5	26
Large exercises	4	6
Legitimacy as a capacity	2	5
Marking	2	8
Medical knowledge	5	12
Motivated and eager	2	3
Real missions	4	15
Regional or national capacity	4	9
Safety	5	25
Training	5	20
Trust	5	21
USAR experience in other missions	1	2
Work environment	1	4

**Fig. 1:**
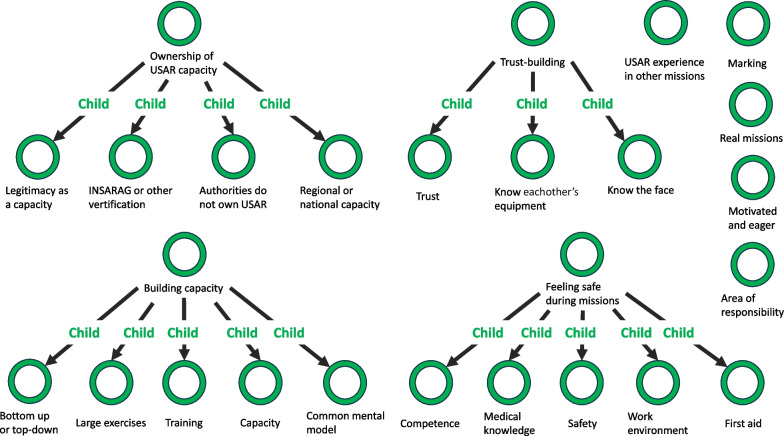
Phase 3: structure of codes, probable themes

**Fig. 2:**
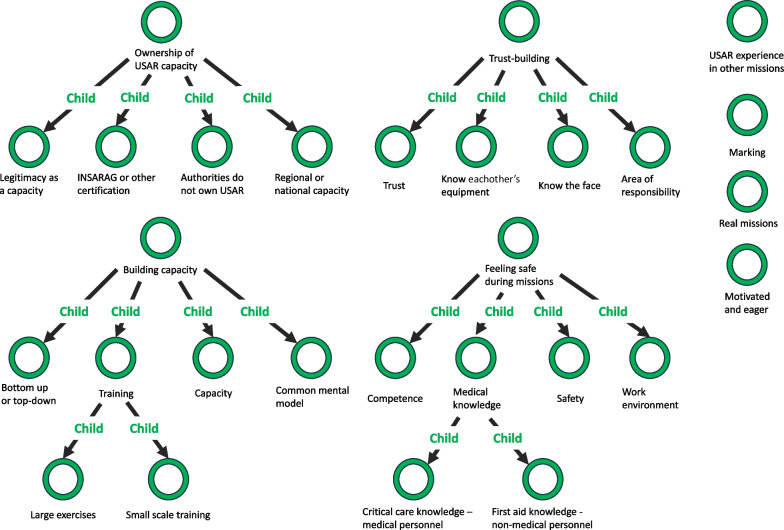
Phase 4: reviewed themes

**Fig. 3:**
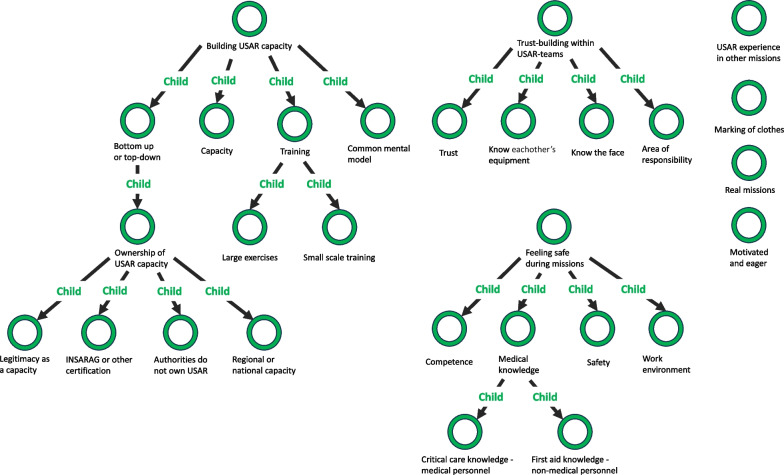
Phase 5 revised

• Phase 3—search for themes (Fig. 1)

• Phase 4—reviewing themes (Fig. 2)

• Phase 5—defining and naming themes (Fig. 3)

**Fig. 4:**
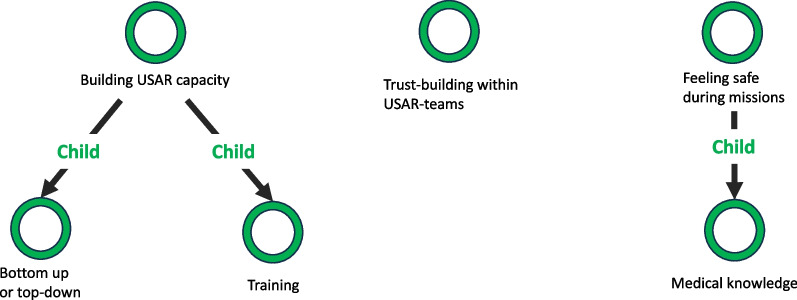
Phase 5: Finally defined themes

This final step is refining the codes into workable themes and end with Fig. 4.

Phase 6—writing the report

### Approval

Application for approval of this research project was submitted October 2’nd 2022 to Norwegian Agency for Shared Services in Education and Research with reference number 838900. Consent was received October 24’th 2022.

As no patient information, health information from participants or any other information subject to protection under Norwegian law is gathered, approval from Regional Committees for Medical and Health Research Ethics was not needed.

## Results

This study aimed to identify how USAR trained EMS-personnel contribute, and how can they offer even better assistance to both rescuers and victims in USAR-incidents as perceived by USAR-trained police officers and firefighters. The three themes identified were: “Feeling safe during missions” (what EMS-personnel contribute), “Trust-building within USAR teams” (what contributes to bilateral trust between operators from different agencies), and “Building USAR capacity” (what it takes from the involved partners to develop a USAR capacity). All the quotes in this article are translated from Norwegian by EW and verified by MH.

### Feeling safe during missions

The interviewees seem to realize that their own annual training in basic lifesaving techniques (e.g., cardiopulmonary resuscitation, lateral position and stopping catastrophic external bleedings) is not enough to manage injuries in a USAR-environment. They value having medical professionals as part of the team in the immediate vicinity.

“I noticed when we found the first victim [at Gjerdrum [12]], and I didn’t quite remember who BE was there and then, but they were ready and came into this pretty complicated landslide with their gear. [ … ] Especially if the patient was alive, we didn’t know that just then. And then they were there, and I felt: “Ahh… It’s good to have them here!”

*Police officer, eight years experience*

*“Again, it means a lot for us and our safety that they are there. It was a scary place.”*

*Firefighter, ten years experience (about the landslide at Gjerdrum)*

Throughout all interviews, the interviewees reported that they appreciate the fact that USAR trained health professionals are nearby and, because of their training and equipment, are able to work effectively even in the hazardous area.

The goal of prehospital services in Norway is “bringing the hospital to the patient” [13]. Considering this, the respondents reported that they feel reassured that they will not be expected to assess, prioritize, and start treating patients in a dangerous accident site; they are happy to leave this to USAR-trained health professionals.

“Stabilizing a patient if there is life in there, that’s something they are far better at than we are. If we were to be competent on that too, we would have to train less on other stuff, so…”

*Firefighter, three years experience*

On the other hand, several respondents valued cooperative theoretic and practical training, and expressed interest in learning more about medical patient treatment than they currently receive in their own service.

“My point is: with those Styrofoam sheets with quick clay sloshing on both sides, if someone topside had said to me: “Yes, [first name], bring your dog. Let’s balance 250 metres into the landslide area to the top of that smashed house you can see over there”. I would never have done it. But because I had health expertise and fire and rescue service with me, my risk assessment was that the risk is acceptable. That’s my thought about the matter.”

*Police officer, twelve years experience*

### Trust-building within USAR-teams

The participants often mentioned that mutual trust is paramount. Not only trust in personnel’s competence within their profession and their ability to be part of a cross-agency team in complex environments, but also trust on a more personal level. Those that have worked together with BE in real missions experienced and regarded BE’s members as trustworthy contributors. Many feel a combination of basic training, smaller exercises, real assignments, or just social meeting points outside work contributes to trust-building.

“Coming up topside on the crane seeing there are two from BE there with their harnesses on, means I know they are checked out, that they trust my work, and I trust them. When we’re all rigged up, and they are ready to climb over the rail, they just: “Yes!” without hesitation.”

*Firefighter, three years experience*

In all the interviews, the most mentioned phenomena regarding trust is the importance of recognizing a face. Having an open dialogue when discussing solutions, methods, prioritization, or simply sharing knowledge is often mentioned as easier when the firefighters and police officers meet familiar faces from the ambulance service.

“Hi, nice to see you again! Let’s get to work together!”

*Police officer, fifteen years experience*

“I know from experience that things are easier when we just say: “Hi, nice to see you again!”, and then crack on with the job at hand.”

*Firefighter, ten years experience*

Respondents from both agencies in all interviews report that they relax meeting a familiar face from BE, because the natural feeling of uncertainty about mutual trust and competence in a high-risk operation are put aside.

All the USAR-certified dogs in OPD are patrol dogs, meaning they normally are tools to manage unwilling individuals or crowds and to search for missing individuals. In police terminology, patrol dogs are referred to as weapons.

“What we saw up there [at Gjerdrum] was that OBRE is very aware our dogs are also weapons. They move calmly and in a circle around us, while several from BE looked like they were thinking “rescue dog!” and came right up to greet. So, we had to explain that: “Yes, sort of, but not right now.” This would have been best clarified in advance.”

*Police officer, twelve years experience*

According to findings in this study, mutual trust and belief in each other’s competence are building blocks for inter-agency operations. This is an example that this trust easily can be compromised, and that BE needs more insight in Victor’s tools (the dogs) and their methods.

### Building USAR capacity

USAR Oslo has built a USAR emergency capability based on selected and volunteer operators from three different agencies. This capacity has been built bottom up [14], meaning no directive from political or directorate level has been issued. The technical and theoretical competence needed has come from the ground level, almost solely from OBRE. OPD and BE have accepted the courses given by OBRE and have implemented their selection system for operators.

“The word out is that it’s only a matter of time before a major landslide occurs in Oslo, there are areas full of quick clay where there’s potential for a slide. If you then have no preparedness, you have to use those that know a little. We make ourselves better if we know more than just a little. And that we have trained together. […] That’s preparedness.”

*Firefighter, three years experience*

All three agencies have hours set aside for training. The overall impression is that cross-agency training is paramount and more of it is desired. Respondents identified two routes to developing competence: “training” and “large-scale exercises”. Training is described as low-key, focused on technical skills and cooperation with informal debriefs and discussion around details. Large-scale exercises comprise all three agencies and include a plan for the exercise with specific goals, controllers to evaluate, and a formal debrief to establish learning points for all participants. Large-scale exercises thus can be said to be a test of preparedness and capacity, not a training platform for building those.

“Yes, please, both of them. We have to practice separately, because we have our own stuff to take care of too but would also like very much to train more together. That’s my opinion.”

*Firefighter, ten years experience*

“Distinguish between practicing but also have exercises in the form of a case. We will train first, then be sent to an unknown object and maybe spend the day, doing several incidents. That will give a better outcome.”

*Firefighter, three years of experience*

Several respondents expressed pride in what USAR Oslo has evolved into. Firefighters and police officers with normally entirely different work tasks seemed to value contributing with their individual expertise in collaborative effort. Many expressed that they expect from the authorities to outline national preparedness for USAR incidents, preferably based on USAR Oslo.

## Discussion

The findings suggest firefighters and police officers have overall positive experiences and opinions on working with ambulance personnel in USAR. This aligns very well with INSARAG Guidelines [2] and two other extensive publications on building USAR capacity [4, 5] that support the need for medical professionals in USAR teams.

Working as police officers and firefighters is inherently risky. Both training and actual work exposes such personnel to physical and psychological threats that they seek to minimize both individually and through organizational measures.

Both police officers and firefighters seem to realize that injuries sustained in a USAR-setting can be more complicated than their own training prepares them for. They also express relief when ambulance personnel take responsibility for assessing and treating patients, even those that have not experienced it themselves report that patients are best handled by health professionals. This opinion can be based on the principles from Norwegian authorities on managing large incidents [1] or their own experiences or fears of having to manage a field of expertise they feel they do not master.

Feeling safe as part of a team working in a dangerous environment necessitate trust [15]. Informants emphasized the need for mutual trust, suggesting that even tough emergency workers feel the need for safety, especially in dangerous situations. They also express the need and appreciation for being trusted back. This fundamental human need to trust and feel trusted seems to be paramount within emergency services and should not be underestimated [15]. Sharing knowledge contributes to developing trust, but trust is also a prerequisite for sharing knowledge [16, 17].

In recent years, establishing a shared mental model between emergency personnel has had an increasing focus [1, 18–21]. A shared mental model is easy to achieve within one service of people with the same education and the same view on how to interpret and reach their goals. However, among personnel from different agencies, it is less straightforward. In all five interviews, the need for more cross-agency training was expressed without anyone expressing the opposite. Thus, the general assumption among USAR-specialists is that effectively establishing shared mental model on high-risk assignments depends on practicing together, and that this is a prerequisite for effective rescue operations. A typical example for this need is the quote about search dogs also being weapons. The police officer felt that USAR personnel from BE did not take the necessary precaution around a search dog at work. It is reasonable to presume that this is because BE and Victor have not trained enough together for them to trust BE operators understand fully that police patrol dogs at work need to be handled with great care, and that others should keep their distance as a general rule unless invited.

The development of USAR Oslo is not based on an assignment or project established by any governmental entity or top management in any of the three agencies. Ground level special rescue professionals in OBRE with knowledge about and interest in USAR reached out to Victor in 2015. This type of development could be described as bottom-up [14]. In all five interviews concern is expressed about the lack of focus from superior entities on a national or regional level.

Indeed, the lack of governmental ownership of USAR preparedness is often mentioned. The prevailing opinion seems to be that the future of USAR in Norway depends on the governmental support. Specifically, governmental bodies need to give all three emergency services the assignment and funding necessary to build a robust USAR organization comprised of education, training, and governance. Firefighters in particular reported that the responsibility for such a task delegated to each local fire and rescue service would be impossible, as most services in Norway are too small to be able to maintain such a specialized capacity. The police and ambulance service would meet the same challenge in rural areas. Several participants in the interviews express that specialized rescue capacities such as USAR would best be established on a regional or national basis.

There is a feeling of pride within USAR Oslo among the interviewees that emerges across diverse comments and answers. A special source of pride is the fact that the governmental commission’s evaluation after the Gjerdrum landslide concluded the success of the acute effort in the first hours was crucial for preventing loss of lives [12]. Many reported that USAR Oslo has proven their ability to deliver, especially at Gjerdrum, in close cooperation with USAR-trained personnel from Nedre Romerike Brann & Redning. Despite such experienced and documented success, there is a lack of directorial or political initiative to give the field focus and development opportunity. As such, respondents reported disappointment, pessimism, and even hopelessness.

### Relevance

To our knowledge, no one has ever asked firefighters and police officers what they have experienced and think about working closely with ambulance personnel in precarious situations. As USAR incidents are rare and always unique, an explorative study seems to be most appropriate. We thus undertook a qualitative study to gather data from a variety of personnel, where factors like experience, exposure, and preconceptions among the informants could add colour and depth to the canvas. To ensure efficiency in data gathering, focus groups interviews was selected instead of individual interviews. The group dynamic effect among colleagues who know each other very well could also lead to more openness, honesty and sharing of creative opinions [7], which was desired.

### Validity and transferability

The selection of contributors in the focus groups has been entirely up to the services discretion. The criteria were that they were certified USAR-operators within their service, willing, and accepting that the interviews were taped and used in an article. Internal validity [22, 23] is addressed by review of the interview guide after each interview and by thoroughly following the six phase model of the method [10, 24].

The size of focus groups is a possible limitation to our study. Malterud describes a project should comprise three to five interviews, each with six to ten participants [6]. Two of the interviews in this study has only three participants, the other three has four. Reading the results should take this into account. There is a possibility that those not interviewed have other opinions on the topics covered in the interviews, but no hints of rumours or organizational opinions to the contrary of findings in the collected data emerged during the interviews. On this basis we regard the results as representative for the two USAR-trained colleges at OBRE and Victor.

The findings in this study are directly valid to other services and regions in Norway, and to other countries with similar organizational boundaries between emergency services. External validity wise this can be understood as that the opinions and psychosocial mechanisms uncovered, like trust, familiar faces, the value of social encounters, small training sessions with informal debriefs, are probably valid in a broader area of creating teams across organizational lines. We have not investigated how USAR collaboration is organized in other countries, but it is known that there are many variations, where both privately funded non-for-profit organizations and governmental emergency services may have the capacity for both domestic and international USAR efforts. For instance, USAR health capacity in Great Britain is funded over the national budget through National Ambulance Resilience Unit (NARU), in Canada national and regional capacities are funded and organized by Public Safety Canada via Disaster Financial Assistance Arrangements (DFAA). The European Union has funded partially or in full the establishment of USAR capacities in several European countries, and representatives from several Norwegian fire and rescue departments have recently attended conferences and courses in their process.

#### Reflexivity

Reflexivity is the question of how our own assumptions and biases influenced this research project. EW works as a paramedic in the air ambulance department at Oslo University Hospital, with ten years’ experience as USAR-specialist. He is also a CBRNe (Chemical, Biological, Radiation, Nuclear and explosions) specialist in the ambulance department and is a familiar face among USAR-specialists in all three agencies. It is fair to be aware of possible bias in favour of USAR Oslo. To counter this possibility, MH conducted the interviews. He had no insight into USAR, nor is he a familiar face to USAR specialists in OBRE and Victor. Furthermore, themes have been identified and highlighted based on the number of times statements have been coded and the sincerity with which they have been voiced. Finally, EWs preconceptions about the need for USAR preparedness in Norway and the model for building a capacity similar to USAR Oslo is supported by United Nations [2], the evaluation report after the quick clay landslide at Gjerdrum [12], and two extensive master thesis [4, 5]. These preconceptions are not controversial; indeed they are aligned with the demand that emergency services collaborate in complex rescue missions, something Norwegian authorities have regarded as a prerequisite [1, 25].

## Conclusions

The strong feeling of mutual trust within the USAR Oslo community and the expressed need for medical expertise in the dangerous area of USAR incidents, has led to EMTs and paramedics being perceived as an integrated and natural part of USAR efforts. The USAR operators feel they are part of a tightly knit “special emergency family”, where hierarchy and colour on the uniform is of lesser importance.

The main findings suggest small scale training sessions builds competence, develops techniques, and builds trust, while full scale exercises are suitable for proving capacity.

## Data Availability

The datasets used and/or analysed during the current study are available from the corresponding author on reasonable request.

## Abbreviations

BE: Beredskapsenheten (preparedness unit, part of the ambulance service at Oslo University Hospital)
CBRNe: Chemical, biological, radiation, nuclear, explosions
EMCC: Emergency medical call centre
EMS: Emergency medical service
EMT: Emergency medical technician (basic ambulance education level)
GP: General practitioner
INSARAG: International search and rescue advisory group
OBRE: Oslo Brann & Redningsetat (Oslo fire & rescue service)
OCHA: Office for the coordination of humanitarian affairs
NORSAR: Norwegian search and rescue
UHP: USAR health professional
UN: United Nations
USAR: Urban search and rescue
Victor: Callsign and “street name” for the Oslo Police District patrol dog unit
